# Xanthine Oxidase Inhibitor, Febuxostat Is Effective against 5-Fluorouracil-Induced Parotid Salivary Gland Injury in Rats Via Inhibition of Oxidative Stress, Inflammation and Targeting TRPC1/CHOP Signalling Pathway

**DOI:** 10.3390/ph15020232

**Published:** 2022-02-16

**Authors:** Walaa Yehia Abdelzaher, Mohamed A. Nassan, Sabreen Mahmoud Ahmed, Nermeen N. Welson, Gaber El-Saber Batiha, Hanaa Mohamed Khalaf

**Affiliations:** 1 Department of Pharmacology, Faculty of Medicine, Minia University, Minia 61519, Egypt; walaayehia22@yahoo.com (W.Y.A.); hanaa_16.5m12@yahoo.com (H.M.K.); 2 Department of Clinical Laboratory Sciences, Turabah University College, Taif University, P.O. Box 11099, Taif 21944, Saudi Arabia; m.nassan@tu.edu.sa; 3 Department of Human Anatomy and Embryology, Faculty of Medicine, Minia University, Minia 61511, Egypt; Sabreen.ahmed@deraya.edu.eg; 4 Department of Basic Medical Sciences, Faculty of Physiotherapy, Deraya University, New Minia City 61768, Egypt; 5 Department of Forensic Medicine and Clinical Toxicology, Faculty of Medicine, Beni-Suef University, Beni Suef 62511, Egypt; 6 Department of Pharmacology and Therapeutics, Faculty of Veterinary Medicine, Damanhour University, Damanhour 22511, Egypt; gaberbatiha@gmail.com

**Keywords:** febuxostat, fluorouracil, parotid damage, CHOP, TRPC1

## Abstract

The current research aimed to examine the ameliorative role of febuxostat (FEB), a highly potent xanthine oxidase inhibitor, against 5-fluorouracil (5-FU)-induced parotid salivary gland damage in rats, as FEB is a pleiotropic drug that has multiple pharmacological effects. A total of 32 Wistar adult male rats were randomly arranged into four groups. Group 1: the control group; given only the vehicle for 14 days, then given a saline i.p. injection from the 10th to the 14th day. Group 2: the FEB group; rats received FEB (10 mg/kg) once daily po for 14 days before receiving a saline i.p. injection from the 10th to the 14th day. Group 3: the 5-FU group; from the 10th to the 14th day, rats received an intraperitoneal injection of 5-FU (35 mg/kg/day). Group 4: the FEB/5-FU group; rats were pre-treated with FEB po for 14 days before receiving 5-FU i.p injections for five consecutive days from the 10th to the 14th day. Parotid gland damage was detected histologically and biochemically by the evaluation of oxidative stress markers (malondialdehyde (MDA) and nitric oxide levels (NOx)), oxidant defences (reduced glutathione (GSH) and superoxide dismutase (SOD)), inflammatory markers (tumour necrosis factor-alpha (TNF-α), interleukin-1β (IL-1β)), and transient receptor potential canonical1 (TRCP1) and C/EBP homologous protein (CHOP). FEB pre-treatment reduced MDA, TNF-, and IL-1 while increasing SOD, GSH, and NOx. FEB also significantly increased TRPC1 and decreased CHOP in parotid gland tissue. In conclusion, FEB pre-treatment reduced 5-FU-induced parotid salivary gland damage not only through its powerful anti-inflammatory and antioxidant effects, but also through its effect on the TRPC1/CHOP signalling pathway.

## 1. Introduction

Chemotherapeutic drugs are needed to treat cancer patients all over the world [1]. Regrettably, they have several harmful effects on both normal and malignant cells, which are injured through a variety of pathways associated with the stomatognathic system’s key structures and functional activities [2,3]. One of these chemotherapeutic medicines is fluorouracil (5-FU), which is used to treat solid malignancies such as breast, GIT, ovarian, and brain tumours [4]. 5-FU is a pyrimidine analogue that blocks the incorporation of thymidylate synthase into RNA. Through its enzymatic barrier, it also inhibits nitrogenous base synthesis and DNA, interrupting the cell cycle [3]. 5-FU is a cytostatic drug that is used in combination with calcium leucovorin (LV) to treat colorectal cancer. Rieko and his colleagues [5] found that the 5-FU + LV therapy damaged the salivary glands in rats. In addition, 5-FU caused deleterious pathological changes in the salivary glands and tongue mucosa [4].

One of the side effects of radiotherapy and chemotherapy is oral mucositis, which increases the length of hospitalisation, expense, and survival rates in cancer patients. Acute oral soreness, infection, haemorrhage, sepsis, bacteraemia, salivary gland atrophy, and xerostomia are all consequences that might impair nutritional intake, salivary gland functions, body weight, and oral hygiene in cancer patients, especially in immunocompromised patients [6,7]. Chemotherapy- or radiotherapy-induced mucositis has a complex mechanism that is poorly understood. Mucositis affects around 80% of cancer patients who take 5-FU, with 20% of them experiencing severe mucositis [8]. Anticancer medications can destroy the oral cavity, oropharynx, gut, and skin’s basal epithelium and mucosal cells, as well as stem cells [5].

The efficacy of the available therapies for the treatment of chemotherapy-induced mucositis is still limited. Moreover, most of these drugs do not aim to protect salivary glands against the adverse effects of chemotherapy. Therefore, finding new strategies to alleviate adverse reactions of anticancer agents and to develop novel therapies for mucositis is urgently needed [5].

Specific receptors on the acinar cell plasma membrane of salivary glands are responsible for their stimulation of fluid secretion. This is mediated by an increase in the cytosolic Ca^2+^, which regulates several ion channels and transporters and is considered a ubiquitous second messenger controlling cell functions such as gene expression and cellular homeostasis. The store-operated Ca^2+^ entry (SOCE) mechanism, which is regulated in response to the depletion of endoplasmic reticulum’s (ER) Ca^2+^, determines the sustained Ca^2+^ increase required for prolonged fluid secretion. The major contributor to SOCE and fluid secretion in salivary gland acinar and ductal cells is transient receptor potential canonical1 (TRPC1). The damage of TRPC1 reduces the endoplasmic reticulum’s (ER) Ca^2+^ level and results in the loss of salivary gland cells with an increase in C/EBP homologous protein (CHOP) expression [9,10].

Xanthine oxidase (XO) expression and activity change based on the histologic basis of cancer. Increased XO plasma activity has been linked to an inflammatory response to tissue damage induced by tumour growth in patients with various cancer types. XO can stimulate the metabolic activation of carcinogenic substances, and it can also operate as a tumorigenic agent by producing reactive oxygen and nitrogen species [11]. Irradiation-induced oxidative stress in the salivary glands of rats was demonstrated by a high concentration of xanthine oxidase in the glands, according to Mehmet and colleagues [12]. In a prior study [13], allopurinol was found to reduce chemotherapy-induced stomatitis by blocking xanthine oxidase.

Febuxostat (FEB) is a non-purine-selective xanthine oxidase (XO) inhibitor used to control chronic gout. With the help of superoxide radicals, the XO enzyme produces uric acid. DNA and protein oxidation are caused by reactive oxygen species (ROS). As a result, the protein structure is modified, and functional alterations occur [14]. Oxidative stress has a role in the damage of the salivary glands by chemotherapeutic agents. It affects the antioxidant enzyme defence system, reducing glutathione, and that is accompanied by the interaction of ROS with cellular membranes producing malondialdehyde (MDA) [15]. FEB also has anti-inflammatory properties, as it inhibits TNF-α and IL-6 production in renal, intestinal, and prostatic tissues [14]. On the other hand, FEB has minimal side effects (e.g., nausea, vomiting, abdominal pain, headache, and temporary changes in liver function tests) [16]. Garcia-Valladares reported that FEB is well tolerated by most patients, demonstrating a good safety profile [17]. FEB is a pleiotropic drug that has multiple pharmacological effects, including antioxidant [14], anti-inflammatory [18], anti-fibrotic [19], and anti-angiogenesis [20] functions.

Concerning FEB’s pleiotropic effects, the current study investigated its protective action against 5-FU-induced parotid gland damage, with a focus on its effects on key mediators such as TRPC1, CHOP, tumour necrosis factor-alpha (TNF-α), and interleukin 1 (IL-1β).

## 2. Results

### 2.1. Impact of FEB on the Physical Parameters of 5-FU-Induced Injury of the Parotid Salivary Gland

Data presented in Table 1 show that ΔBW, parotid weight, and parotid index were significantly decreased in rats received 5-FU (35 mg/kg/day) as compared to the corresponding control values. On other hand, rats pre-treated with FEB in a dose of 10 mg/kg showed a significant increase in ΔBW, parotid weight, and parotid index in comparison to the 5-FU group.

### 2.2. Impact of FEB on the Oxidative Stress Parameters of 5-FU-Induced Injury of the Parotid Salivary Gland

Next, we evaluated the effects of FEB (10 mg/kg) on oxidative stress and antioxidant defence in the parotid tissues. The results presented in Table 2 showed that 5-FU significantly increased parotid MDA and NOx levels, which is indicative of increased oxidative stress in 5-FU-induced injury of the parotid salivary gland. On the other hand, the activities of SOD and GSH were significantly decreased in the parotid tissues as compared to the corresponding values in the control. The FEB administration had protective effects against these changes. Contrarily, FEB significantly reduced levels of MDA and NO in the parotid tissues and significantly elevated parotid GSH levels and SOD activity in rats exposed to 5-FU insult.

### 2.3. Impact of FEB on Inflammatory Mediators (TNF-α and IL-1β) of 5-FU-Induced Parotid Salivary Gland Injury

As shown in Table 3, TNF-α and IL-1β protein levels in the parotid tissues were significantly higher in rat given only 5-FU as compared to the corresponding control values. FEB administration prior to 5-FU, on the other hand, resulted in significantly improved inflammatory parameters (TNF-α and IL-1β) when compared to the 5-FU group.

### 2.4. Effect of FEB on TRPC1 and CHOP in 5-FU Induced Parotid Salivary Gland Injury

**Figure 1:**
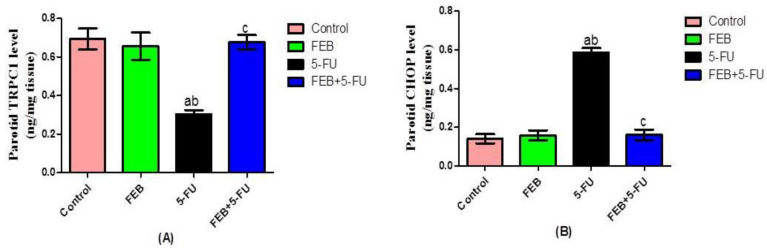
Impact of FEB on TRPC1 (**A**) and CHOP (**B**) in 5-FU-induced parotid salivary gland toxicity. Values are expressed as the mean ± S.D. Results were considered significantly different when *p* < 0.05. ^a^ Significant difference compared to the control group, ^b^ significant difference compared to the FEB group, and ^c^ significant difference compared to the 5-FU group. (TRPC1: transient receptor potential canonical1; CHOP: C/EBP homologous protein; 5-FU: 5-Fluorouracil, and FEB: febuxostat) (8 rats/group).

Rats challenged with 5-FU showed a significant decrease in parotid TRPC1 level (Figure 1A) along with a significant increase in CHOP level in parotid tissue as compared to the corresponding control values (Figure 1A,B). Rats that received FEB before 5-FU showed a significant increase in the parotid TRPC1 level (Figure 1A) with a significant decrease in CHOP level (Figure 1B) when compared to the 5-FU group.

### 2.5. Histological Results

#### 2.5.1. H&E- and Toluidine-Blue-Stained Sections

**Figure 2:**
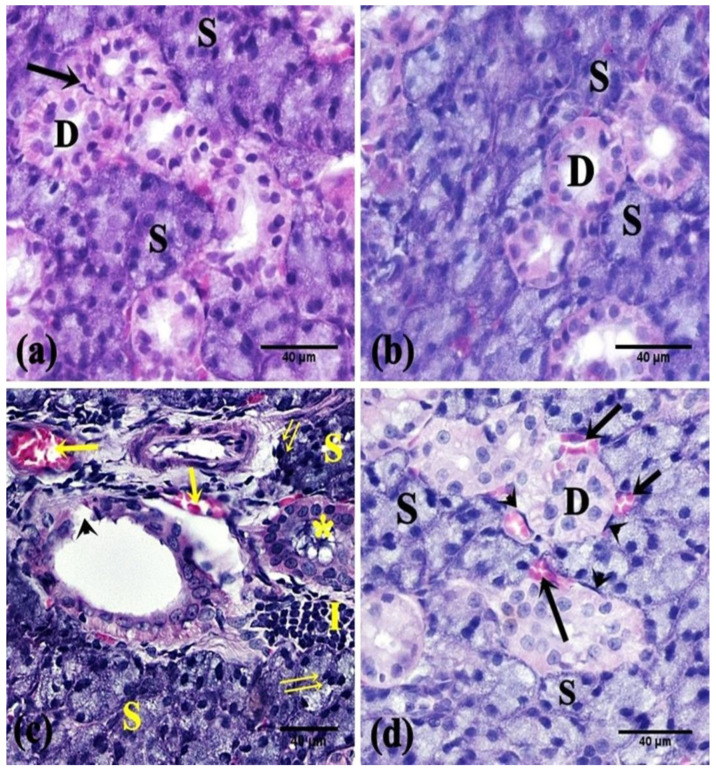
Photomicrographs of the parotid salivary gland of the studied groups. (**a**,**b**) The control and FEB groups, respectively, showed intact parotid parenchyma; serous acini (S) and striated ducts (D). Note the myoepithelial cells (↑). (**c**) The 5-FU group showed parenchymal changes; coalesced serous acini (S), pyknotic nuclei (↑↑), cellular infiltration (I), interrupted lining of striated ducts (arrowhead), retained secretion (*), and dilated congested blood vessels (↑). (**d**) The FEB + 5-FU group showed normal parotid structure; serous acini (S), striated ducts (D), and myoepithelial cells (arrowhead) except for mildly congested blood vessels (↑). (H&E ×400; 8 rats/group).

**Figure 3:**
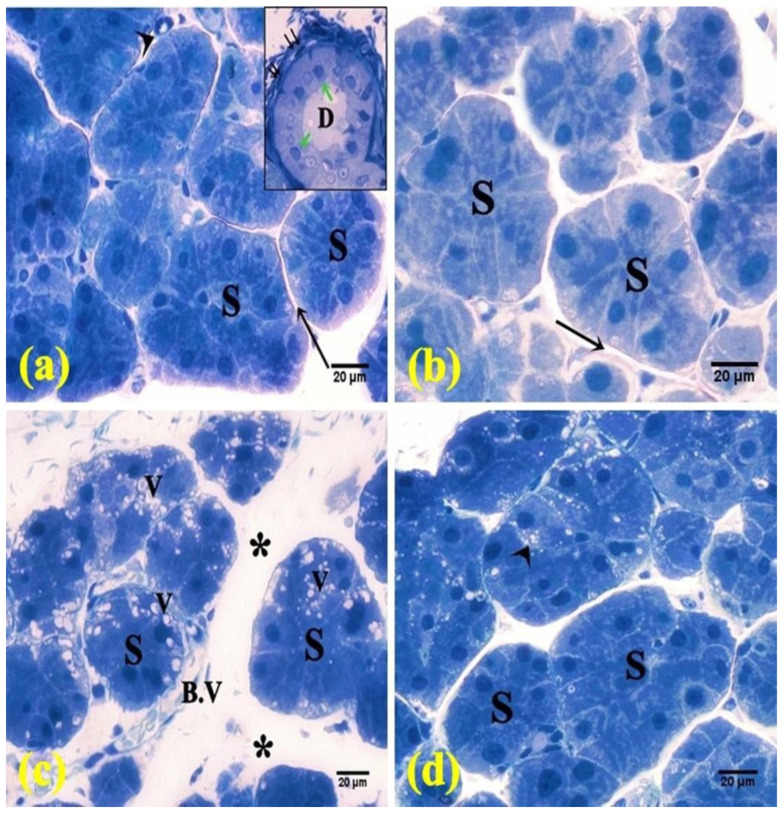
Photomicrographs of semi-thin sections of the parotid salivary gland. (**a**,**b**) The control and FEB groups, respectively, showed intact parenchyma with packed serous acini (S), thin septa (↑), and blood vessel (arrowhead). Inset: striated duct (D) lined by low columnar cells (green arrow) and surrounded with myoepithelial cells (↑↑). (**c**) The 5-FU group showed serous acini (S) separated by wide spaces (*) and congested blood vessel (B.V). Notice the vacuolation (V). (**d**) The FEB + 5-FU group showed packed acini with few vacuolations (arrowhead). (Toluidine blue ×400; 8 rats/group).

H&E-stained sections of the control animals revealed parotid lobules with connective tissue trabeculae in-between. The parenchyma displayed purely packed serous acini in addition to striated ducts. The serous acini were lined by pyramidal cells with basal nuclei. The striated ducts appeared lined by a single layer of cuboidal to low columnar cells with acidophilic cytoplasm, central nuclei, and basal striations (Figure 2a and Figure 3a). In the FEB group, the parotid parenchyma revealed a comparable picture to the control group (Figure 2b and Figure 3b). Meanwhile, the parotid parenchyma sections of the 5-FU rats revealed obvious structural alterations, such as coalesced acini with displaced pyknotic nuclei, cellular infiltration, dilated congested blood vessels, desquamated lining of striated ducts (Figure 2c), wide spaces between the acini, and marked vacuolation (Figure 3c).

On the other hand, the parotid sections of the FEB + 5-FU treated rats revealed almost normal parotid parenchyma with packed arranged acini and striated ducts, except in partial areas with vascular congestion (Figure 2d) and limited vacuolation (Figure 3d).

#### 2.5.2. Immunohistochemically Stained Sections

**Figure 4:**
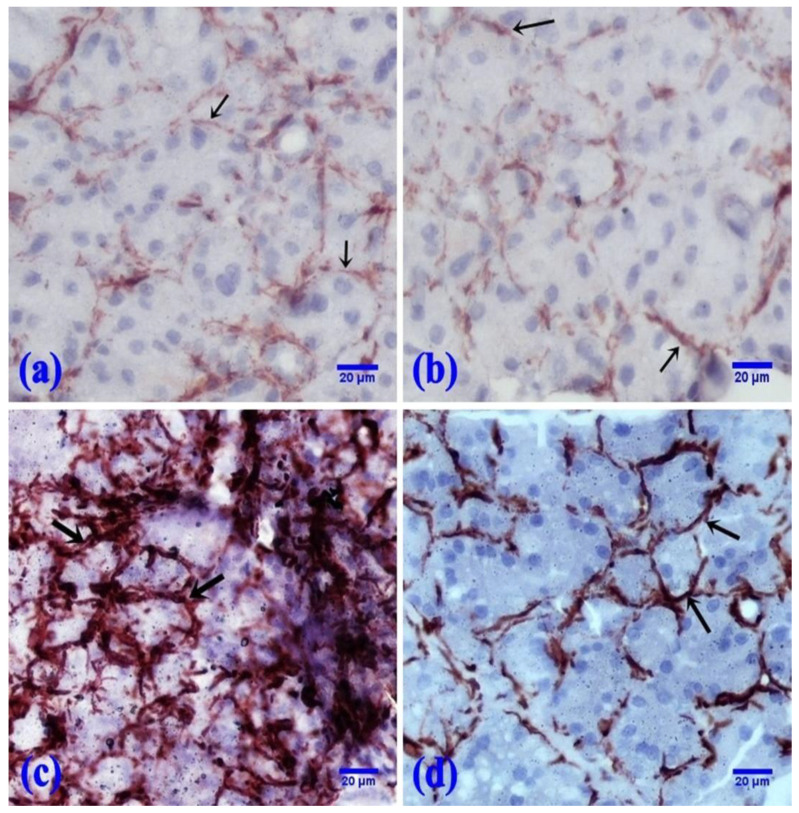
Photomicrographs of the α-SMA immunoreaction at the periphery of the acini (↑). (**a**,**b**) The control and FEB groups, respectively, showed a faint immunoreaction. (**c**) The 5-FU group showed a marked immunoreaction. (**d**) The FEB + 5-FU group showed a moderate immunoreaction. (α-SMA immunostaining, ×400; 8 rats/group).

The control and FEB groups showed a faint immunoreaction for α-SMA at the periphery of the acini (Figure 4a,b). However, 5-FU showed an apparent increase in α-SMA-positive reactions at the periphery of the parotid acini in comparison with the control rats (Figure 4c). Parotid sections of the FEB/5-FU treated rats displayed a moderate immunoreaction to α-SMA at the periphery of the acini (Figure 4d).

#### 2.5.3. Morphometric Results

**Figure 5:**
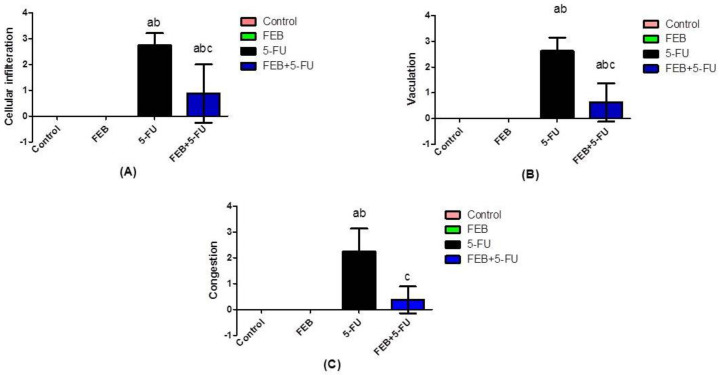
Scores of the histological findings. Values were expressed as the mean ± S.D. (**A**) shows the cellular infiltration score. (**B**) shows the vacuolation score. (**C**) shows the congestion score. Results were considered significantly different when *p* < 0.05. ^a^ Significant difference relative to the control, ^b^ significant difference relative to the FEB group, and ^c^ significant difference relative to the 5-FU group. (5-FU: 5-fluorouracil and FEB: febuxostat; 8 rats/group).

The measured mean area percent for α-SMA expression showed a statistically significant difference between the groups (Table 4). The 5-FU group outscored the other groups in terms of α-SMA expression. On histological examination, the 5-FU group had higher cellular infiltration, vacuolation, and congestion scores than the other groups (Figure 5).

## 3. Discussion

The development of salivary gland damage is dependent on a condition of disequilibrium between oxidative stress, inflammation, and cell death. 5-FU causes this state of imbalance. The majority of the chemotherapeutic agents also pose many challenges to the integrity of the salivary glands [4].

The present data demonstrated that 5-FU-intoxicated rats showed a significant decrease in physical parameters, which was related to parotid salivary gland injury, diarrhoea, and difficulty in swallowing. Alteration of the salivary flow and its components occurred [6,7]. Physical parameters were significantly improved in the FEB-treated rats. Through its antioxidant and anti-inflammatory properties, FEB had pleiotropic pharmacological effects on testosterone-induced benign prostatic hyperplasia and endometrial hyperplasia [14,18].

5-FU enhanced the formation of ROS with superoxide anion (O_2_^−^), consequently triggering salivary glands damage. ROS attack any molecules, mainly cell membranes’ polyenoic fatty acids, forming peroxyl radicals with a chain of reactions of membrane lipid peroxidation and necrosis. MDA, an indicator of lipid peroxidation, was found to be elevated [21]. The termination of ROS damage in healthy cells is complemented by the radical scavenging system, including SOD and GSH [22]. This was in agreement with our results as 5-FU showed significant increases in parotid MDA and NOx levels with decreased parotid SOD and GSH activities as a result of excessive release of ROS and the development of oxidative disequilibrium. Moreover, Bomfin and his colleagues [23] reported that 5-FU induces salivary gland oxidative stress, hindering salivary formation and flow in rats.

FEB effectively limited oxidative tissue injury in various pathological disorders such as benign prostatic hyperplasia [14], endometrial hyperplasia [15], cardiac and bone marrow toxicity [24], cancer [25], and sepsis [26]. As a result, our goal was to investigate FEB’s function in mitigating 5-FU-induced parotid salivary gland damage and to elucidate possible underlying mechanisms.

FEB showed proper antioxidant capabilities, as it greatly reduced the increase in parotid lipid peroxide concentration, NOx, and SOD and GSH deficiency to levels comparable to the control animals. This is in line with FEB’s well-known antioxidant action, which has been shown to protect against a variety of diseases [14]. Through its antioxidant effects, FEB also alleviated hypertension and renal damage [27].

In the current study, 5-FU significantly increased parotid TNF-α and IL-1β levels. Earlier studies had also proven the ability of 5-FU to increase inflammatory mediators. Bomfin et al. [23] reported that 5-FU increased the inflammatory reaction in the salivary glands, allowing the release of pro-inflammatory cytokines with increased inflammatory cells influx. Barbosa and his co-workers [28] showed that 5-FU caused oral mucositis and hyposalivation, leading to a pronounced inflammatory response. In addition, the inflammatory response of 5-FU is linked to the oxidative disequilibrium that enhances the efflux of proinflammatory cytokines through stimulating the redox-sensitive transcription factor [29]. FEB also has anti-inflammatory properties, as it inhibits TNF- α and IL-6 β production in renal, intestinal, and prostatic tissues [14].

In the current study, FEB reduced the levels of such inflammatory mediators significantly, and the histopathological assessment and α-SMA expression testing supported this finding. Some previous reports have demonstrated that FEB has an anti-inflammatory effect. Mohamed and her co-workers [18] found that FEB protected the endometrium against oestrogen-induced endometrial hyperplasia in rats through its anti-inflammatory effect. Similarly, FEB attenuated the development of periodontitis in rats by inhibiting proinflammatory mediators and oxidative stress [14,30].

Calcium is a ubiquitous second messenger that controls cell functions like gene expression and cellular homeostasis. Transient receptor potential canonical 1 (TRPC1) is a channel responsible for the function of the salivary gland. It is involved in the regulation of Ca^2+^ homeostasis. Damage of TRPC1 reduces the endoplasmic reticulum (ER) Ca^2+^ level, resulting in the loss of salivary gland cells with an increase in the C/EBP homologous protein (CHOP) expression [10,31]. The current findings demonstrated a decrease in the parotid TRPC1 with an increase in the CHOP level in the 5-FU group.

Sun et al. [32] found that TRPC1 regulated the Ca^2+^-activated chloride (Cl^−^) channels (CaCC) in the salivary gland cells, as it provided the sustained Ca^2+^ entry, which is important for TMEM16a (CaCC) activation to modulate Cl^−^ efflux. Furthermore, salivary glands diseases and damage are accompanied by disturbances in ER and Ca^2+^ homeostasis. The activation of CHOP with this damage resulted in a decrease in TRPC1 expression, attenuating autophagy and apoptosis, which caused cell death [10].

Consistent with earlier reports from other authors, FEB is a pleiotropic drug that has multiple pharmacological effects, including antioxidant [33], anti-inflammatory [18,30], anti-fibrotic [19], and anti-angiogenesis [20] actions. This is in line with our study as, besides its antioxidant and anti-inflammatory properties, FEB also increased parotid TRPC1 level and decreased parotid CHOP level significantly as compared to the 5-FU group. FEB protected the parotid salivary gland from 5-FU-induced damage through its antioxidant and anti-inflammatory effects, decreasing the disturbances in ER and Ca^2+^ homeostasis and consequently deactivating CHOP and increasing TRPC1 levels.

These current findings were supported by He et al. [34], who reported that FEB attenuated ER stress-mediated renal injury in hyperuricemic nephropathy in rats. On the other hand, FEB at high concentrations had agonistic activity on TRPA1 calcium channels [35].

## 4. Materials and Methods

### 4.1. Drugs & Chemicals

5-FU was obtained from Sigma Co. (Burlington, MA, USA). Febuxostat (FEB) was purchased from Astellas Pharma Philippines Inc., Co. (Yamanouchi, Nagano, Japan). Elabscience Co. (Houston, TX, USA) provided the IL-1β and TNF-α ELISA kits. TRPC1 and CHOP were tested using ELISA kits (Wuhan Fine Biological Technology Co., Wuhan, Hubet, China Catalog No. EH4284 and MyBio Source Co., San Diego, CA, USA, Catalog No. MBS3808179) according to the manufacturer’s instructions.

### 4.2. Animals

A total of 32 adult male albino rats weighing 200–250 gm, aged about 8–10 weeks, were given by the National Research Center (Cairo, Egypt). The rodents were allowed to acclimatise for 1 week before the experiment. The maintenance conditions were 25 ± 2 °C temperature, a dark/light cycle of 12 h, and free intake of tap water and regular rat chow (El-Nasr Co., Cairo, Egypt). Procedures involving animals’ care adhered to the ARRIVE guidelines and followed the U.K. Animals Act, 1986. Approval from the board of the Faculty of Medicine, Minia University (Approval No. 41:3/2021), was received.

### 4.3. Experimental Design

#### 4.3.1. Animal Grouping

The animals were randomised into 4 groups (8 rats/group):

Group 1, the control group, was given only the vehicle for 14 days before receiving saline intravenous injections from the 10th to the 14th day. Group 2, the FEB group, received FEB (10 mg/kg) [18] once daily po for 14 days, dissolved in 0.5% carboxymethylcellulose sodium (CMC) [36], and i.p. injections of saline from the 10th to the 14th day. Group 3, the 5-FU group, was injected with 5-FU (35 mg/kg/day) i.p from the 10th to 14th day [5]; 5-FU was dissolved in 1 N NH4OH (50 mg/mL), yielding a clear, colourless to light yellow solution. Group 4, the 5-FU/FEB-treated group, was pre-treated with 10 mg/kg FEB orally for two weeks before receiving 5-FU administration from the 10th to the 14th day. The doses of 5-FU and FEB were justified according to our preliminary experiments and previous studies [5,18,36].

#### 4.3.2. Sampling and Samples Storage

At the end of the experiment, the animals were weighed and slaughtered under anaesthesia with urethane hydrochloride (1 g/kg i.p.). The parotid salivary gland was removed quickly, weighed, and rinsed with saline to eliminate any blood. For histological and immunohistochemical tests, one gland from one side of each rat was excised, fixed in 10% formalin, and embedded in paraffin. The other gland was homogenised in ice-cold phosphate buffer (0.01 M, pH 7.4; 20% *w*/*v*). Tissue pieces were weighed and subsequently homogenised in phosphate buffer with a glass homogeniser on ice (tissue weight (g): phosphate buffer (mL) volume = 1:5). The homogenate was centrifuged for 15 min at 5000 rpm, and the supernatant was stored in multiple microcentrifuge tubes at −80 °C until it was used to measure the biochemical parameters.

### 4.4. Assessment of Physical Indicators

Initial and terminal body weights (wts) were measured at the beginning and end of the experiments. The difference between terminal and initial body weight (ΔBW) was calculated. The ratio of the parotid weight to the total body weight (parotid index) was determined. This index was obtained by dividing the weight of the parotid tissue by the terminal bodyweight of the rat, multiplied by 100.

### 4.5. Biochemical Analysis

#### 4.5.1. Assessment of Oxidative Stress Parameters in the Parotid Salivary Gland

The antioxidant enzymes in the tissue homogenate were measured via measuring superoxide dismutase (SOD) content (U/g tissue) along with the reduced glutathione (GSH) level (μmol/g tissue).

SOD activity was determined chemically according to the method of Marklund and Marklund, 1974 [37], who reported that one unit of SOD is equal to the percent of the enzyme that inhibits the autoxidation of pyrogallol by 50% and measured at 420 nm by spectrophotometry. SOD activity (U/gm tissue) = % inhibition × 3.75 × 1/F. Where F is the weight of parotid per 1 mL of parotid homogenate, which is equivalent to 0.2 g/mL of homogenate.

The reduction of the Ellman’s reagent by thiol (-SH) groups of GSH to create a yellow hue detected at 412 nm was used to determine the GSH level using spectrophotometry [38]. Standards were assayed under the same conditions as tissue samples. The parotid concentration of GSH was measured from the standard curve in µmol/mL, and then it was transferred to µmol/g tissue by the following calculations: GSH (µmol/mL homogenate) × 1/F—where F is the weight of parotid per 1 mL of parotid homogenate, which is equivalent to 0.2 g/mL of the homogenate.

MDA in tissue homogenate was measured using a spectrophotometric approach based on the thiobarbituric acid method, with sample absorption measured at 535 nm [39]. 1,1,3,3-Tetramethoxypropane (TMP) was used to prepare standard concentrations of malondialdehyde (1, 2, 4, 6, 8 and 10 nmol/mL). From this curve, the lipid peroxide concentration in the unknown sample was extrapolated from the corresponding absorbance using the regression line from the standard curve and expressed as nmol/g tissue by multiplying by the tissue dilution factor.

The Griess reaction, which is based on the interaction of nitrite with a combination of naphthyl ethylenediamine and sulfanilamide, was used to determine the total nitrite/nitrate (NOx) in tissue homogenates. At 540 nm [40], NOx levels (nmol/g tissue) were measured. A standard curve was constructed with a set of serial dilutions (from 10 nmol/L to 1 mmol/L) of sodium nitrite. From the curve, the total nitrite content in the unknown sample was extrapolated from the corresponding absorbance using the regression line from the standard curve and expressed as nmol/g tissue.

#### 4.5.2. Assessment of Inflammatory Parameters in the Parotid Salivary Gland

TNF-α and IL-1β levels in tissue homogenate were determined using ELISA kits and the manufacturer’s instructions (Catalog Nos. E-EL-R0012, E-EL-R0019, respectively). These parameters were measured in tissue homogenate and calculated according to the ratio of dilution of the homogenate with lysis buffer of 1:5 (each 1 mL of homogenate contains 0.2 gm of tissue). By multiplying the data by the tissue dilution factor, the results were expressed as Pg/mg of tissue. TNF-α and IL-1β optical densities were measured simultaneously at 450 nm.

#### 4.5.3. TRCP1 and CHOP Levels in the Parotid Salivary Gland

TRCP1 and CHOP levels in tissue homogenates were evaluated using ELISA kits and following the instructions of the manufacturer (catalogue nos. EH4284 and MBS3808179, respectively). In CHOP, the colour in the tubes changed from blue to yellow, and the optical density was read at 450 nm within 15 minutes. In TRCP1, the colour turned yellow immediately, and the optical density was read at 450 nm after adding the stop solution.

### 4.6. Histopathological and Immunohistochemical Examinations

Specimens from the parotid gland were fixed in 10% formalin, processed to produce 5–7 μm thick paraffin slices, and mounted on glass slides for hematoxylin and eosin (H&E) staining [41]. Other slices were placed on positively charged slides and stained with alpha-smooth muscle actin (-SMA) to show myoepithelial cells (MECs) (Glostrup, Denmark: Dako Corporation) [42].

Before being embedded in an epoxy/resin mixture, parotid gland specimens were maintained in 2.5 percent of 0.1 phosphate-buffering glutaraldehyde. Toluidine blue (TB) was used to stain semi-thin (1 mm thick) sections before they were examined under a light microscope [32].

### 4.7. Morphometric Analysis

The image analyser image-j was used to quantify the area percent of the immunopositive expression of α-SMA in five non-overlapping fields from five distinct sections of five different animals in each group at 100 magnifications. The severity of the histopathological changes was graded on a scale of 0 to 3, with 0 indicating no pathologic finding and 1, 2, and 3 indicating pathologic findings in 33, 33–66, and >66 percent of the tissue, respectively [26].

### 4.8. Statistical Analysis

The current experiment statistics are shown as the mean ± standard deviation (S.D). One-way ANOVA was used to examine the data, followed by Tukey’s multiple comparison test. The software GraphPad Prism (v.5) was used. A *p*-value < 0.05 was set for significance.

## 5. Conclusions

The presented data revealed that FEB is a powerful remedy with the capability to battle comorbidities in parotid salivary gland damage. FEB exhibited beneficial effects in regulating the TRPC1/CHOP signalling pathway. It had strong antioxidant properties, inhibited the effects of IL-1β and TNF-α, and modulated the expression of α-SMA. Future studies are needed to investigate the efficacy of FEB with various dose profiles, either alone or as adjuvant therapy to cure parotid salivary gland damage conditions.

## Figures and Tables

**Table 1:** Impact of FEB on the physical parameters (ΔBW, parotid wt, and parotid index) of 5-FU-induced injury of the parotid salivary gland in rats.

Groups	ΔBW	Parotid wt (gm)	Parotid Index (%)
Control	18.38 ± 5.854	0.34 ± 0.09	10 ± 0.0
FEB	16.88 ± 4.35	0.32 ± 0.64	10 ± 0.0
5-FU	10.00 ± 7.42 ^ab^	0.07 ± 0.02 ^ab^	3.3 ± 0.39 ^ab^
FEB + 5-FU	17.63 ± 5.29 ^c^	0.29 ± 0.076 ^c^	8.6 ± 0.84 ^c^

Data are shown as their mean ± S.D (8 rats/group). ^a^ Significant difference from the control group, ^b^ significant difference from the FEB group, and ^c^ significant difference from the 5-FU group. (ΔBW: the difference between the terminal and initial body weights, 5-FU: 5-fluorouracil, and FEB: febuxostat.).

**Table 2:** Impact of FEB on oxidative stress parameters (MDA, SOD, GSH, and NOx) in 5-FU-induced injury of the parotid salivary gland in rats.

Groups	MDA (nmol/g Tissue)	SOD (U/g Tissue)	GSH (μmol/g Tissue)	NOx (nmol/g Tissue)
Control	8.53 ± 1.08	371 ± 18.9	23.25± 2.61	7.42 ± 0.76
FEB	8.73 ±0.86	339 ± 39.2	22.54 ± 1.24	7.26 ± 0.87
5-FU	17.56 ± 1.45 ^ab^	149 ± 16.75 ^ab^	17.81 ± 1.51 ^ab^	16.08 ± 0.71 ^ab^
FEB + 5-FU	9.62 ± 0.30 ^c^	339 ± 45.95 ^c^	22.83 ± 1.44 ^c^	10.80 ± 1.67 ^c^

The data are shown as the mean ± S.D (8 rats/group). ^a^ Significant difference relative to the control rats, ^b^ significant difference relative to the FEB group, and ^c^ significant difference compared to the 5-FU group. (MDA: malondialdehyde; SOD: superoxide dismutase; GSH: reduced glutathione; NOx: total nitrite/nitrate; 5-FU: 5-fluorouracil and FEB: febuxostat).

**Table 3:** Impact of FEB on inflammatory parameters (TNF-α and IL-1β) of 5-FU-induced parotid salivary gland injury in rats.

Groups	TNF-α (Pg/mg Tissue)	IL-1β (Pg/mg Tissue)
Control	46.23 ±3.24	22.42 ±1.72
FEB	49.06 ±4.34	22.93 ± 1.9
5-FU	78.02 ± 7.01 ^ab^	41.23± 4.76 ^ab^
FEB + 5-FU	51.87 ± 2.08 ^c^	23.50 ±1.87 ^c^

The data are shown as the mean ± S.D (8 rats/group). ^a^ Significant difference from the control animals, ^b^ significant difference relative to the FEB treated rats, and ^c^ significant difference compared to the 5-FU group.

**Table 4:** Changes in area% of α-smooth muscle actin.

Groups	Area% of α-SMA
Control	2.19 ± 1.28
FEB	2.16 ± 1.06
5-FU	9.26 ± 1.94 ^ab^
FEB + 5-FU	4.53 ± 1.03 ^abc^

Values are expressed as the mean ± S.D (8 rats/group). ^a^ Significant difference from the control rats, ^b^ significant difference from the FEB group, and ^c^ significant difference from the 5-FU group; *p* < 0.05. (α-SMA: alpha smooth muscle actin; 5-FU: 5-fluorouracil; and FEB: febuxostat).

## Data Availability

All data are fully available and included in the manuscript.

## References

[1] Ferlay J., Soerjomataram I., Dikshit R., Eser S., Mathers C., Rebelo M., Parkin D.M., Forman D., Bray F. (2015). Cancer Incidence and Mortality Worldwide: Sources, methods and major patterns in GLOBOCAN 2012. Int. J. Cancer.

[2] Hafez S.M.N.A., Elbassuoni E., Abdelzaher W.Y., Welson N.N., Batiha G.E.-S., Alzahrani K.J., Abdelbaky F.A.F. (2021). Efficacy of vitamin E in protection against methotrexate induced placental injury in albino rats. Biomed. Pharmacother..

[3] Bachmeier E., López M.M., Linares J.A., Brunotto M.N., Mazzeo M.A. (2019). 5-Fluorouracil and Cyclophosphamide modify functional activity in submandibular gland of rats. J. Oral Res..

[4] Elmansy M.N., Hegazy E.M. (2020). Evaluation of the Apoptotic changes induced by 5-Fluorouracil on the Lingual Mucosa and Salivary glands of male albino rats (Histological, Histomorphometric and Immunohistochemical Study). Egypt. Dent. J..

[5] Fujiwara R., Harada K., Ferdous T., Mishima K. (2022). Amino Acids May Have Protective Effects on Salivary Glands of 5-FU-administered Mice. In Vivo.

[6] Epstein J.B., Thariat J., Bensadoun R.J., Barasch A., Murphy B.A., Kolnick L., Popplewell L., Maghami E. (2012). Oral complications of cancer and cancer therapy: From cancer treatment to survivorship. CA Cancer J. Clin..

[7] Murphy B.A. (2007). Clinical and economic consequences of mucositis induced by chemotherapy and/or radiatiotherapy. J. Support. Oncol..

[8] Symonds R.P. (1998). Treatment-induced mucositis: An old problem with new remedies. Br. J. Cancer.

[9] Kania E., Pajak B., Orzechowski A. (2015). Calcium homeostasis and ER stress in control of autophagy in cancer cells. BioMed Res Int..

[10] Sukumaran P., Sun Y., Zangbede F.Q., da Conceicao V.N., Mishra B., Singh B.B. (2019). TRPC1 expression and function inhibit ER stress and cell death in salivary gland cells. FASEB Bioadv..

[11] Emiliya C., Mariyana A., Rossen B., Katarina T., Zaklina S., Adriana B., Andrija S. (2020). 3′-Methyl-4-thio-1H-tetrahydropyranspiro-5′-hydantoin platinum complex as a novel potent anticancer agent and xanthine oxidase inhibitor. Arch. Pharm..

[12] Mehmet A., Seyithan T., Elif B., Elif D., Hilal A., Muslum A., Habip B., Zeynel A.K. (2017). Radioprotective effect of thymoquinone on salivary gland of rats exposed to total cranial irradiation. Head Neck.

[13] Nakamura K., Natsugoe S., Kumanohoso T., Shinkawa T., Kariyazono H., Yamada K., Baba M., Yoshinaka H., Fukumoto T., Aikou T. (1996). Prophylactic action of allopurinol against chemotherapy-induced stomatitis—inhibition of superoxide dismutase and proteases. Anti-Cancer Drugs.

[14] Abdel-Aziz A.M., El-Tahawy N.F.G., Haleem M.A.S.A., Mohammed M.M., Ali A.I., Ibrahim Y.F. (2020). Amelioration of testosterone-induced benign prostatic hyperplasia using febuxostat in rats: The role of VEGF/TGFβ and iNOS/COX-2. Eur. J. Pharmacol..

[15] Yousef D.M., Abd El-Fatah S.S., Al-Semeh M.D., Amira E. (2019). Oxidative Stress Changes Induced by Methotrexate on Parotid Gland Structure of Adult Male Albino Rat: Can Vitamin C Ameliorate These Changes?. Med. J. Cairo Univ..

[16] Love B.L., Barrons R., Veverka A., Snider K.M. (2010). Urate-Lowering Therapy for Gout: Focus on Febuxostat. Pharmacother. J. Hum. Pharmacol. Drug Ther..

[17] Garcia-Valladares I., Khan T., Espinoza L.R. (2011). Efficacy and safety of febuxostat in patients with hyperuricemia and gout. Ther. Adv. Musculoskelet. Dis..

[18] Mohamed M.Z., El Baky M.F.A., Hassan O.A., Mohammed H.H., Abdel-Aziz A.M. (2020). PTEN/PI3K/VEGF signaling pathway involved in the protective effect of xanthine oxidase inhibitor febuxostat against endometrial hyperplasia in rats. Hum. Exp. Toxicol..

[19] Inoue M.-K., Yamamotoya T., Nakatsu Y., Ueda K., Inoue Y., Matsunaga Y., Sakoda H., Fujishiro M., Ono H., Morii K. (2018). The Xanthine Oxidase Inhibitor Febuxostat Suppresses the Progression of IgA Nephropathy, Possibly via Its Anti-Inflammatory and Anti-Fibrotic Effects in the gddY Mouse Model. Int. J. Mol. Sci..

[20] Bir S.C., Kolluru G.K., McCarthy P., Shen X., Pardue S., Pattillo C.B., Kevil C.G. (2012). Hydrogen Sulfide Stimulates Ischemic Vascular Remodeling Through Nitric Oxide Synthase and Nitrite Reduction Activity Regulating Hypoxia-Inducible Factor-1α and Vascular Endothelial Growth Factor–Dependent Angiogenesis. J. Am. Heart Assoc..

[21] Refaie M.M., Shehata S., Bayoumi A., El-Tahawy N.F.G., Abdelzaher W.Y. (2021). The IL-6/STAT Signaling Pathway and PPARα Are Involved in Mediating the Dose-Dependent Cardioprotective Effects of Fenofibrate in 5-Fluorouracil-Induced Cardiotoxicity. Cardiovasc. Drugs Ther..

[22] Gulcin I., Beydemir S. (2013). Phenolic compounds as antioxidants: Carbonic anhydrase isoenzymes inhibitors. Mini Rev. Med. Chem..

[23] Bomfin L.E., Braga C.M., Oliveira T.A., Martins C.S., Foschetti D.A., Santos A.A., Costa D.V., Leitão R.F., Brito G.A. (2017). 5-Fluorouracil induces inflammation and oxidative stress in the major salivary glands affecting salivary flow and saliva composition. Biochem. Pharmacol..

[24] El-Sheikh A., Abdelzaher W., Gad A., Abdel-Gaber S. (2020). Purine versus non-purine xanthine oxidase inhibitors against cyclophosphamide-induced cardiac and bone marrow toxicity in rats. Hum. Exp. Toxicol..

[25] Mangerich A., Dedon P., Fox J.G., Tannenbaum S.R., Wogan G.N. (2013). Chemistry meets biology in colitis-associated carcinogenesis. Free Radic. Res..

[26] Devrim E., Avc A., Ergüder I.B., Karagenç N., Külah B., Durak I. (2008). Activities of Xanthine Oxidase and Superoxide Dismutase Enzymes in Rat Intestinal Tissues in Sepsis. J. Trauma Inj. Infect. Crit. Care.

[27] Takahiro M., Akihiro S., Lusi X., Jiahe Q., Asako N.-T., Yoshiko O., Masahiro K., Osamu I. (2022). Febuxostat amliorates high salt intake-induced hypertension and renal damage in Dahl salt sensitive rats. J. Hypertens..

[28] Barbosa S., Pereira V., Wong D., Santana A., Lucetti L., Carvalho L., Barbosa C., Callado R., Silva C., Lopes C. (2019). Amifostine reduces inflammation and protects against 5-fluorouracil-induced oral mucositis and hyposalivation. Braz. J. Med. Biol. Res..

[29] Haddad J.J. (2002). Redox regulation of pro-inflammatory cytokines and IκB-α/NF-κB nuclear translocation and activation. Biochem. Biophys. Res. Commun..

[30] Nessa N., Kobara M., Toba H., Adachi T., Yamamoto T., Kanamura N., Pezzotti G., Nakata T. (2021). Febuxostat Attenuates the Progression of Periodontitis in Rats. Pharmacology.

[31] Liu X., Ong H.L., Ambudkar I. (2018). TRP Channel Involvement in Salivary Glands—Some Good, Some Bad. Cells.

[32] Ahmed S.M., Fouad F.E. (2019). Possible protective effect of platelet-rich plasma on a model of cisplatin-induced nephrotoxicity in rats: A light and transmission electron microscopic study. J. Cell. Physiol..

[33] Sun Y., Birnbaumer L., Singh B.B. (2015). TRPC1 regulates calcium-activated chloride channels in salivary gland cells. J. Cell. Physiol..

[34] He L., Fan Y., Xiao W., Chen T., Wen J., Dong Y., Wang Y., Li S., Xue R., Zheng L. (2017). Febuxostat attenuates ER stress mediated kidney injury in a rat model of hyperuricemic nephropathy. Oncotarget.

[35] Mihai D., Ungurianu A., Ciotu C., Fischer M., Olaru O., Nitulescu G., Andrei C., Zbarcea C., Zanfirescu A., Seremet O. (2021). Effects of Venlafaxine, Risperidone and Febuxostat on Cuprizone-Induced Demyelination, Behavioral Deficits and Oxidative Stress. Int. J. Mol. Sci..

[36] Ran J., Xu G., Ma H., Xu H., Liu Y., Tan R., Zhu P., Song J., Lao G. (2017). Febuxostat Attenuates Renal Damage besides Exerting Hypouricemic Effect in Streptozotocin-Induced Diabetic Rats. Int. J. Nephrol..

[37] Marklund S., Marklund G. (1974). Involvement of the Superoxide Anion Radical in the Autoxidation of Pyrogallol and a Convenient Assay for Superoxide Dismutase. Eur. J. Biochem..

[38] Moron M., Depierre J., Mannervik B. (1979). Levels of glutathione, glutathione reductase and glutathione S-transferase activities in rat lung and liver. Biochim. Biophys. Acta (BBA)-Gen. Subj..

[39] Buege J., Aust S. (1978). Microsomal lipid peroxidation. Methods Enzymol..

[40] Ridnour L.A., Sim J.E., Hayward M.A., Wink D.A., Martin S.M., Buettner G., Spitz D. (2000). A Spectrophotometric Method for the Direct Detection and Quantitation of Nitric Oxide, Nitrite, and Nitrate in Cell Culture Media. Anal. Biochem..

[41] Abdelzaher W.Y., Ahmed S.M., Welson N.N., Alsharif K.F., Batiha G.E., Labib D.A. (2021). Dapsone Ameliorates Isoproterenol-Induced Myocardial Infarction via Nrf2/HO-1; TLR4/TNF-α Signaling Pathways and the Suppression of Oxidative Stress, Inflammation, and Apoptosis in Rats. Front. Pharmacol..

[42] El-Naseery N.I., Elewa Y.H.A., Ichii O., Kon Y. (2018). An experimental study of menopause induced by bilateral ovariectomy and mechanistic effects of mesenchymal stromal cell therapy on the parotid gland of a rat model. Ann. Anat.-Anat. Anz..

